# One‐Year Outcome of Intensive Insulin Therapy Combined to Glucose‐Insulin‐Potassium in Acute Coronary Syndrome: A Randomized Controlled Study

**DOI:** 10.1161/JAHA.117.006674

**Published:** 2017-11-14

**Authors:** Wahid Bouida, Kaouthar Beltaief, Mohamed Amine Msolli, Nasri Bzeouich, Adel Sekma, Malek Echeikh, Malek Mzali, Hamdi Boubaker, Mohamed Habib Grissa, Riadh Boukef, Mohsen Hassine, Zohra Dridi, Asma Belguith, Fadhel Najjar, Ines Khochtali, Semir Nouira, the GREAT Network

**Affiliations:** ^1^ Emergency Department Fattouma Bourguiba University Hospital Monastir Tunisia; ^2^ Emergency Department Sahloul University Hospital Sousse Tunisia; ^3^ Hematology Department Fattouma Bourguiba University Hospital Monastir Tunisia; ^4^ Cardiology Department Fattouma Bourguiba University Hospital Monastir Tunisia; ^5^ Department of Preventive Medicine Fattouma Bourguiba University Hospital Monastir Tunisia; ^6^ Biochemistry Department Fattouma Bourguiba University Hospital Monastir Tunisia; ^7^ Department of Internal Medicine and Endocrinology Fattouma Bourguiba University Hospital Monastir Tunisia; ^8^ Research Laboratory LR12SP18 University of Monastir Tunisia

**Keywords:** acute coronary syndrome, glucose‐insulin‐potassium, intensive insulin therapy, pharmacology, prognosis, Acute Coronary Syndromes, Mortality/Survival, Complications, Restenosis

## Abstract

**Background:**

A number of factors may offset the cardioprotective effects of glucose‐insulin‐potassium (GIK) on outcome of patients with acute coronary syndrome, such as hyperglycemia induced by this cocktail infusion. We performed a study to evaluate the effect of intensive insulin therapy in association with GIK on 1‐year outcome in patients hospitalized for acute coronary syndrome.

**Methods and Results:**

In a randomized prospective controlled trial we included 772 patients with non–ST‐segment elevation acute coronary syndrome. Patients were randomized into 3 groups: GIKI_2_ group, who received GIK with intensive insulin therapy for 24 hours; GIK group, who received GIK with nonintensive insulin therapy; and control group, who received usual care. The primary outcome criteria were the rates of major cardiovascular events combining death, reinfarction, and stroke rate at 1 year. In addition, we measured platelet function assay‐100 and plasminogen activator inhibitor‐1 at admission and 24 hours later. Based on an intention‐to‐treat analysis, major cardiovascular events at 1 year was 12.8% in the GIKI_2_ group, 15.5% in the GIK group, and 20.5% in the placebo group; the difference was significant between the GIK_2_ and control groups (*P*=0.01). Platelet function assay‐100 at 24 hours decreased significantly from baseline in the control group but not in the GIKI_2_ group. Plasminogen activator inhibitor‐1 decreased significantly in the GIKI_2_ group but significantly increased in the control group. Minor hypoglycemic events were more frequent in the GIKI_2_ group compared with other groups.

**Conclusions:**

GIKI_2_ led to improvement of 1‐year outcome rates in patients with non–ST‐segment elevation acute coronary syndrome. This beneficial effect was associated with a decrease in platelet reactivity and an increase on fibrinolysis tests.

**Clinical Trial Registration:**

URL: https://www.clinicaltrials.gov. Unique identifier: NCT00965406.

**Clinical Perspective**

**What Is New?**

- Potential benefit from glucose‐insulin‐potassium infusion in patients with acute coronary syndrome may be offset by the detrimental effects of hyperglycemia.
- We found that in patients with non–ST‐segment elevation acute coronary syndrome, tight glycemic control with insulin is associated with a decrease of major cardiovascular adverse events at 1 year compared with glucose‐insulin‐potassium infusion alone or standard therapy.

**What Are the Clinical Implications?**

- The potential benefit of glucose‐insulin‐potassium infusion combined to intensive glycemic control in non–ST‐segment elevation acute coronary syndrome supports the need for reappraisal of glucose‐insulin‐potassium infusion therapy and warrants further investigation.

An improvement in clinical outcome of acute coronary syndrome (ACS) may be achieved by therapy strategies using metabolic modulation. Finding effective treatment with cardioprotective effects is more profoundly required than ever with regard to the increasing incidence of coronary artery disease. Administration of intravenous glucose, insulin, and potassium (GIK) was proposed in the treatment of acute myocardial infarction to promote metabolic myocardial protection.^1^, ^2^ The potential mechanisms by which GIK could improve clinical outcomes in ACS includes the decrease of the amount of circulating free fatty acids while enhancing the use of glucose as the primary energy substrate for myocardial tissue.^3^, ^4^ Although early trials have shown some benefit of GIK treatment,^5^, ^6^, ^7^, ^8^, ^9^ other studies have not demonstrated such benefit.^2^, ^10^, ^11^, ^12^, ^13^, ^14^ It was suggested that the inability of GIK to improve outcome might partly be attributable to the tendency of GIK to increase serum glucose levels.^15^, ^16^ In fact, through its deleterious effects on oxidative stress, inflammation, and coagulation, hyperglycemia is considered one of the most important prognostic predictors of early outcome in ACS.^17^, ^18^, ^19^, ^20^ Viewed in this context, a strategy using intensive insulin therapy to avoid GIK‐induced hyperglycemia would be beneficial in patients with ACS. The objective of this study was to compare the effect of intensive insulin therapy associated with GIK compared with GIK alone and with usual care on major cardiovascular events at 1‐year follow‐up in patients with non–ST‐segment elevation ACS.

## Patients and Methods

This was a prospective, randomized, controlled, open‐label study performed between August 2010 and June 2014. We included patients older than 30 years who were admitted to the emergency department for ACS. Diagnosis of ACS was based on pain characteristics, clinical examination, ECG findings, and results of cardiac ischemic markers, according to American College of Cardiology/American Heart Association recommendations.^21^ Patients were eligible for inclusion if they presented with non–ST‐segment elevation with ischemic symptoms lasting ≥10 minutes without persistent ST‐segment elevation on ECG and had at least one of the following: ischemic ECG changes (≥2 leads demonstrating ST depression ≥0.5 mV, T‐wave inversion ≥2 mV, or transient ST‐segment elevation ≥1 mm), elevated cardiac troponin, or history of coronary artery disease (previous myocardial infarction, coronary revascularization, or positive stress test). Individuals with ST‐segment elevation ACS and those with contraindications for GIK infusion including those with type 1 diabetes mellitus or those with known hyperkalemia at randomization were excluded. We also excluded patients with acute heart failure or acute pulmonary edema and patients with renal failure (blood creatinine >200 μmol/L). The study conformed to clinical practice guidelines and followed the recommendations of the Helsinki Declaration. The local ethics review board approved the protocol. Written informed consent was obtained from all patients before enrollment. To ensure that almost equal numbers of patients received each of the 3 treatments, a computer‐based randomization block was used. Patients were randomly assigned to receive the following: (1) An infusion through a peripheral venous access catheter of 500 mL of glucose 10% with 50 IU of rapid‐acting insulin and 70 mmol of potassium at a volume rate of 1.5 mL/kg per hour within 24 hours (GIK group). In this group, regular insulin therapy using a subcutaneous route every 4 hours was administered to maintain a target capillary blood glucose level between 120 and 180 mg/dL. (2) GIK infusion (500 mL) combined to intensive intravenous insulin therapy (GIKI_2_ group). The target level of glycemia in this group was set between 80 and 110 mg/dL. (3) Usual care consisting of subcutaneous insulin therapy similar to that of the GIK group (control group) without GIK infusion.

For the 3 groups, blood glucose determinations (Accu‐Chek Active, Roche) were performed every hour during the 24‐hour protocol. Serum glucose, potassium, and sodium levels were measured at baseline and at 6 and 24 hours after randomization (Dade Behring). To ensure good acceptance, during the 3 months preceding the start of the study, all nurses were trained by a week‐long series of 1‐hour in‐service training sessions and all acquired very good experience with the infusion protocol related to intensive insulin therapy. Blood samples were also performed at baseline and 24 hours later to assess platelet function assay 100 (PFA‐100) according to the manufacturer's instructions. Plasma fibrinolysis status was assessed by plasminogen activator inhibitor‐1 (PAI‐1) activity using the synthetic chromogenic method and a colorimetric assay (Stachrome PAI‐1, Diagnostica Stago). Because the level of PAI‐1 activity is characterized by a diurnal variation, fasting blood samples were taken during the early morning. Intra‐assay variation was ±5%. Biologic assays were performed without knowledge of the allocated group. All patients received conventional treatment of ACS according to current recommendations including antiplatelet agents, anticoagulants, and angiotensin‐converting enzyme inhibitors. We recorded demographic and presenting data including vital signs, test results pertaining to ACS, emergency department care, blood glucose level monitoring, and clinical outcome to list major cardiovascular adverse events (MACE) including death, reinfarction, and stroke. Reinfarction was defined as a new cardiac ischemic event 72 hours from the index admission. Stroke was defined as unequivocal signs of focal or global neurological deficit of sudden onset and a duration of 24 hours that were judged to be of vascular origin. Patients were contacted to obtain information regarding their clinical outcome 30 days and 1 year following discharge. If the patient was not contactable, the next of kin and/or the patient's general practitioner were contacted. Where no information could be obtained, a request was made to the Department of Births and Deaths to ascertain whether the patient was deceased. Physicians who collected outcome data were not aware of treatment allocation at the time that they made the determination. The primary end point was 1‐year rate of combined MACE. Any event associated with death was counted as death, otherwise only the first occurrence event was counted. Secondary end points were 24‐hour variation from baseline of PFA‐100 and PAI‐1. Hypoglycemic episodes were recorded during the protocol. Hypoglycemia was defined as a blood glucose <3.0 mmol/L and was recorded as with or without symptoms.

### Statistical Analysis

We analyzed all data according to the intention‐to‐treat principle. The Kolmogrov‐Smirnov test was used to evaluate whether the variables were normally distributed. Data are presented as mean (SD) or median interquartile range for continuous variables and number with percentage for categorical variables. Student *t* test or Wilcoxon rank‐sum test was used to analyze the statistical significance of differences as appropriate. For global testing of significance, chi‐square test was used for categorical outcomes and Kruskal–Wallis test was used for nonparametric outcomes. Two group comparisons were performed with the chi‐square test, unless the number of acute adverse events was <5, in which case, Fisher exact test was used. For each treatment, results were further expressed as odds ratios, with corresponding 95% confidence intervals, relative to the control group. A 2‐sided *P*<0.05 was considered statistically significant. Analyses were performed using commercial software (SPSS Inc). The sample size was calculated to demonstrate a reduction in the primary end point of 1‐year MACE from 20% in the control group to 10% in the GIKI_2_ group. We estimated that a total of 750 randomized patients (250 in each groups) were needed to detect this difference with a power of 80% and a significance level of 0.05 (2‐sided). If we consider an overall 3% loss to follow‐up, the resulting required sample size would be 772 (257 in each group). This trial is registered with ClinicalTrials.gov number NCT00965406.

## Results

**Figure 1:**
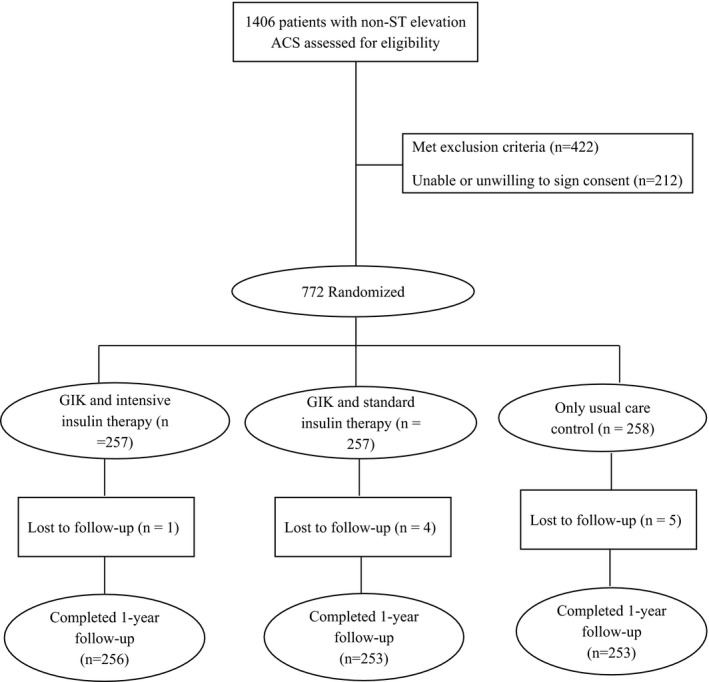
Flow of participants through the GIKI_2_ (Glucose‐Insulin‐Potassium With Insulin Therapy vs GIK Alone) trial. ACS indicates acute coronary syndrome; GIK, glucose‐insulin‐potassium.

**Figure 2:**
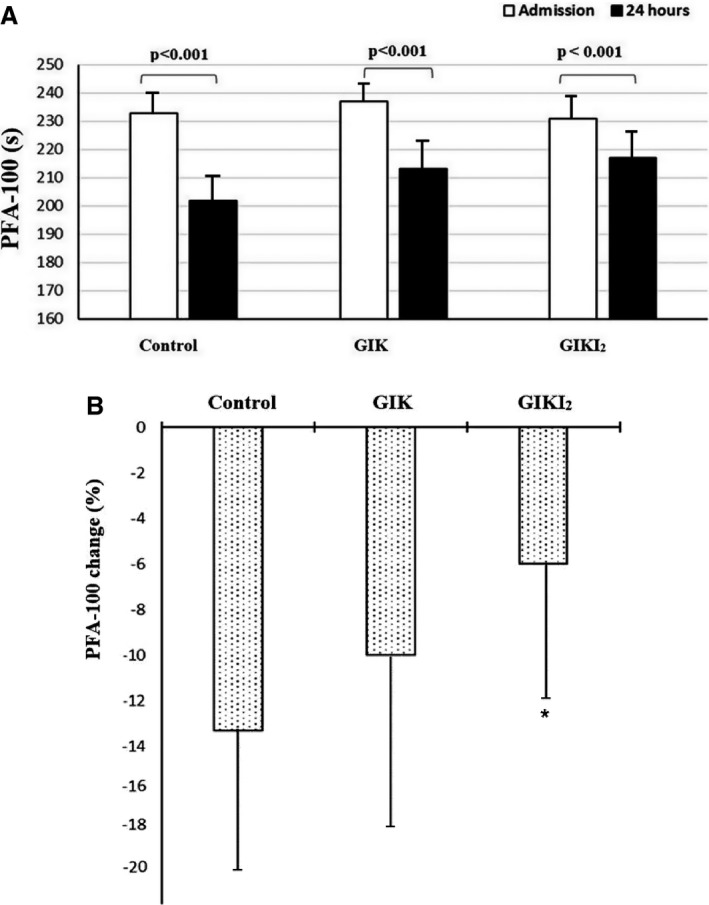
A, Mean platelet function assay‐100 (PFA‐100) values at hospital admission and 24 hours later in the control group (usual care), the glucose‐insulin‐potassium (GIK) group, and the GIK with intensive insulin therapy (GIKI_2_) group. B, Percentage of PFA‐100 variation between hospital admission and 24 hours later in the control group, the GIK group, and the GIKI_2_ group. *P=0.014 vs the control group.

**Figure 3:**
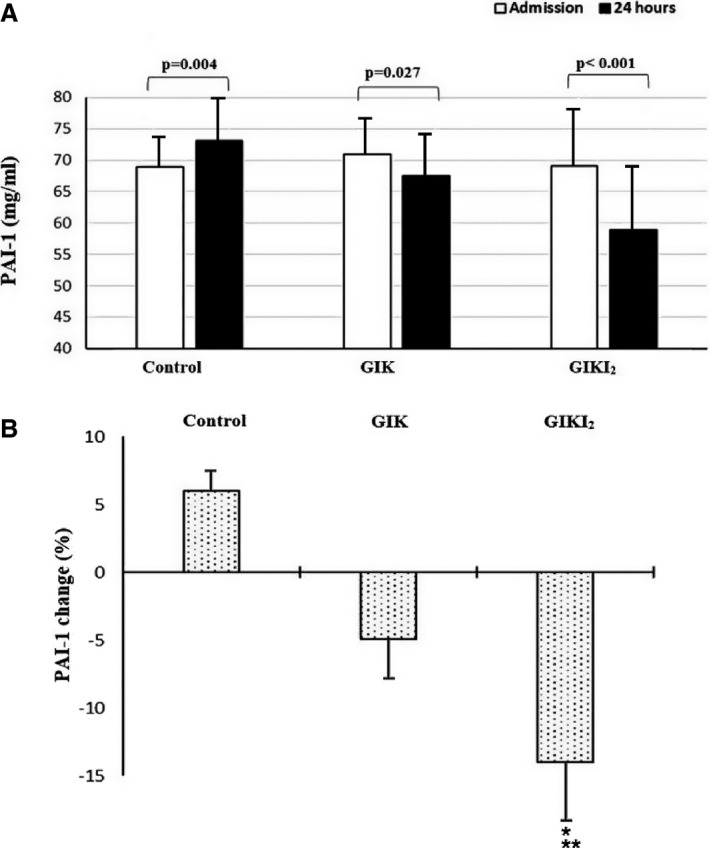
A, Mean plasminogen activator inhibitor‐1 (PAI‐1) values at hospital admission and 24 hours later in the control group (usual care), glucose‐insulin‐potassium (GIK) group, and the GIK with intensive insulin therapy (GIKI_2_) group. B, Percentage of PAI‐1 variation between hospital admission and 24 hours later in the control group, GIK group, and GIKI_2_ group. *P<0.001 vs the control group **P<0.001 vs the GIK group.

We screened 1406 patients, of whom 772 provided informed consent to participate in the trial (Figure 1). A total of 422 patients met exclusion criteria and 212 patients were unable or unwilling to sign consent. Demographic characteristics and clinical variables at admission of this last group were not significantly different compared with the overall population included in the trial. Enrolled participants were randomly assigned to 1 of the 3 study groups: control (n=258), GIK (n=257), and GIKI_2_ (n=257). At emergency department admission, the 3 groups were comparable with regard to age, sex, medical history, and the class of risk based on the thrombolysis in myocardial infarction risk score (Table 1). Follow‐up data were not obtained from 10 participants (5 in the control group, 4 in the GIK group, and 1 in the GIKI_2_ group). Six patients moved away from the study site during the trial and the others (n=4) were unreachable. Glucose goals that were targeted by our algorithm were achieved in 70.9% of the GIKI_2_ group. Mean glucose level during the 24‐hour protocol period was 8.1±4.8 mmol/L in the GIKI_2_ group compared with 12.3±4.9 mmol/L in the GIK group and 11.9±3.3 mmol/L in the control group (*P*<0.01). Table 2 shows the outcomes stratified by treatment group. In the intention‐to‐treat population, the MACE rate at 30 days was 6.2% (n=16) in the control group, 7.0% (n=18) in the GIK group, and 3.1% (n=8) in the GIKI_2_ group. The difference was not statistically different across the 3 groups (*P*=0.12). One‐year MACE occurred in 53 patients (20.5%) in the control group, 40 patients (15.5%) in the GIK group, and 33 patients (12.8%) in the GIKI_2_ group. The 1‐year MACE rate was significantly different between the GIKI_2_ and control groups (odds ratio, 0.56; 95% confidence interval, 0.36–0.91 [*P*=0.01]) but was not significantly different between the GIK and control groups (odds ratio, 0.71; 95% confidence interval, 0.45–1.12 [*P*=0.14]). The 1‐year MACE rate did not differ significantly between the GIKI_2_ and GIK groups (odds ratio, 0.79; 95% confidence interval, 0.49–1.31 [*P*=0.37]). All individual components of MACE at 30 days and 1 year were lower in the GIKI_2_ group compared with the other groups; however, the difference was statistically significant only for reinfarction rate (Table 2). PFA‐100 values were not significantly different at admission between the 3 groups (235±48 seconds versus 237±37 seconds versus 233±42 seconds for the GIKI_2_, GIK, and control groups, respectively; *P*=0.26). After 24 hours, PFA‐100 decreased significantly in the 3 groups (Figure 2A). The relative decrease of the PFA‐100 was significantly lower in the GIKI_2_ group (−6%) compared with the control group (−13.3%, *P*=0.01) but not significantly different between the GIKI_2_ and GIK groups, nor between the GIK and control groups (Figure 2B). PAI‐1 values at admission were similar in the 3 groups (*P*=0.61). PAI‐1 values increased in the control group at 24 hours (+6%) but decreased in the GIK and GIKI_2_ groups (−4.9% and −14.7%, respectively). All of these changes from baseline were significant within each group (Figure 3A). Compared with the control group, the percentage of change from baseline in PAI‐1 was significantly different in both intervention groups (Figure 3B). The decrease of PAI‐1 at 24 hours was more significant in the GIKI_2_ group compared with the GIK group (*P*<0.001). Hypoglycemic events were more frequent among the GIKI_2_ group compared with the GIK group (8.3% versus 1.9%; *P*<0.01). No hypoglycemic events were reported in the control group. Symptomatic hypoglycemic events were observed only in the GIKI_2_ group (n=1).

**Table 1:** Baseline Demographic and Clinical Characteristics of the Intention‐to‐Treat Population

Characteristic	Control Group (n=258)	GIK Group (n=257)	GIKI_2_ Group (n=257)
Age, y	62±10	62±11	62±10
Male/female ratio	2.03	2.16	2.09
Cardiovascular risk factors, No. (%)			
Hypertension	140 (54.2)	131 (50.9)	142 (55.2)
Diabetes mellitus	125 (48.4)	134 (52.1)	134 (52.1)
Dyslipidemia	71 (27.5)	67 (26.0)	51 (23.7)
Coronary artery disease	113 (43.8)	100 (38.9)	95 (36.9)
Current smoker	123 (47.6)	120 (46.7)	113 (43.9)
Medication use before admission, No. (%)			
Aspirin	166 (64.3)	151 (58.7)	160 (62.2)
Clopidogrel	40 (15.5)	42 (16.3)	38 (14.8)
Diuretics	73 (28.3)	84 (32.7)	71 (27.6)
ß‐Blockers	67 (25.9)	59 (22.9)	56 (21.8)
Oral antidiabetics	73 (28.3)	76 (29.5)	89 (34.6)
Insulin	52 (20.1)	57 (22.1)	45 (175)
Lipid‐lowering agents	96 (37.2)	103 (40.0)	98 (38.1)
ACEIs/ARBs	39 (15.1)	44 (17.1)	37 (14.4)
Systolic blood pressure, mm Hg	148±26	152±31	160±60
Diastolic blood pressure, mm Hg	81±51	83±19	88±35
Heart rate, beats per min	77±16	83±21	83±20
TIMI risk score	2.6±1.3	2.6±1.5	2.6±1.2
Laboratory data			
Baseline glucose, mmol/L	2.0±0.9	1.8±0.9	1.9±0.7
Creatinine clearance, mL/min	78.1±32.2	75.2±30.6	75.9±29.5
Platelet count, 1000/mm^3^	204.2±81.2	239±76.3	218.1±44.6
Troponin I, median (IQR), mg/mL	30.7 (5.9–81.1)	31.1 (9.9–63.8)	33 (10.1–48.0)

ACEIs indicates angiotensin‐converting enzyme inhibitors; ARBs, angiotensin receptor blockers; GIK, glucose‐insulin‐potassium; GIK_2_, GIK with intensive insulin therapy; IQR, interquartile range; TIMI, thrombolysis in myocardial infarction. Values are expressed as mean±SD unless otherwise indicated. Assessed by the Cockcroft‐Gault formula.

**Table 2:** Clinical Outcome in the Intention‐to‐Treat Population

	Control Group (n=258)	GIK Group (n=257)	GIKI_2_ Group (n=257)	GIK vs Control Group OR (95% CI)	*P* Value	GIKI_2_ vs Control Group OR (95% CI)	*P* Value
Reinfarction, No. (%)							
30 d	7 (2.7)	8 (3.1)	1 (0.4)^a^	1.15 (0.35–3.79)	0.78	0.14 (0.02–0.85)	0.04
y	25 (9.7)	13 (5.0)	11 (4.2)	0.49 (0.25–0.98)	0.04	0.41 (0.21–0.85)	0.019
Stroke, No. (%)							
30 d	1 (0.4)	1 (0.4)	0 (0)	···	···	···	···
1 y	4 (1.5)	3 (1.1)	3 (1.1)	0.75 (0.17–3.38)	0.70	0.75 (0.17–3.38)	0.70
Death, No. (%)							
30 d	8 (3.1)	9 (3.5)	7 (2.7)	1.13 (0.38–3.43)	0.79	0.87 (0.26–2.80)	0.79
1 y	24 (9.3)	24 (9.3)	19 (7.4)	1.00 (0.52–1.90)	1	0.77 (0.39–1.52)	0.43
Combined MACE, No. (%)							
30 d	16 (6.2)	18 (7.0)	8 (3.1)	1.13 (0.56–2.32)	0.71	0.48 (0.21–1.14)	0.09
1 y	53 (20.5)	40 (15.5)	33 (12.8)^b^	0.71 (0.45–1.12)	0.14	0.56 (0.36–0.91)	0.019

CI indicates confidence interval; GIK2, glucose‐insulin‐potassium (GIK) with intensive insulin therapy; MACE, major cardiovascular events; OR, odds ratio.

a *P*<0.05 vs the control and GIK groups.

b *P*<0.05 vs the control group.

## Discussion

In this prospective, randomized, open‐label, controlled trial of patients with non–ST‐segment elevation ACS, intensive insulin therapy combined with GIK for 24 hours reduced the rate of MACE at 1 year compared with GIK alone or usual care. Death and stroke occurred less frequently in the GIKI_2_ group but the difference was not significant. The 1‐year MACE rate decreased from 20.5% under usual care (control group) to 12.8% with GIKI_2_, which means that the number needed to treat to prevent one MACE was ≈13 patients. This clinical benefit was associated with a significant decrease in PFA‐100 and increase in PAI‐1 used respectively as markers of platelet reactivity and fibrinolysis inhibition. No major hypoglycemic events were reported.

For many years it has been well demonstrated that elevation of blood glucose levels during ACS is associated with an increase in short‐ and long‐term mortality and hospital morbidity.^21^ Hyperglycemia has prothrombotic and proinflammatory effects and is associated with endothelial dysfunction.^22^, ^23^, ^24^ It was suggested that there is probably a link between hyperglycemia and the lack of efficacy of GIK infusion in patients with ACS. Therapy with GIK has long been prescribed during the acute phase of myocardial infarction, but available data on its benefit have been controversial.^2^, ^5^, ^6^, ^7^, ^8^, ^9^, ^10^, ^11^, ^12^, ^13^, ^14^ In the DIGAMI (Diabetes Mellitus, Insulin Glucose Infusion in Acute Myocardial Infarction) 1 study^6^ where GIK administration was associated with aggressive glucose lowering, a significant reduction in 1‐year mortality was demonstrated in patients with ST‐segment elevation myocardial infarction.^4^ However, 2 other large studies, the DIGAMI 2 trial^25^ and CREATE‐ECLA‐II (Clinical Trial of Metabolic Modulation in Acute Myocardial Infarction Treatment Evaluation‐Estudios Cardiologicos Latinoamerica)^12^ did not confirm such beneficial effect of GIK therapy in similar patients. The common factor in both negative studies is that glycemic levels were not closely controlled. Moreover, in the first DIGAMI study, an increase in serum glucose concentration was observed in the GIK infusion group compared with the control group at 6 and 24 hours after treatment, which raises the possibility that higher serum glucose level with GIK infusion may have blunted the potential benefits of insulin. By maintaining euglycemia, insulin would lead to the protection of ischemic myocardium in ACS and improvement in outcome. In their study on the molecular side of glucose regulation in myocardial infarction, Marfella et al^26^ concluded that glycemic control can reduce remodeling and apoptosis of peri‐infarcted areas in patients with ACS by reducing oxidative stress and inflammation. In a complementary analysis of the CREATE‐ECLA study that showed a lack of benefit from GIK infusion, Chaudhuri et al^16^ suggested that ≈18% in mortality could be reduced if GIK‐induced hyperglycemia was avoided by insulin therapy. In our study, blood glucose levels were not different between the GIK and control groups, which could argue against the hypothesis that GIK failure is caused by induced hyperglycemia. Our explanation to this result is the fact that we used a low glucose concentration (10% glucose), which may cause little elevation of glycaemia. In addition, the sample size in our study was calculated on the basis of a 1‐year MACE rate and could be smaller than required to show a significant difference of glycemia between GIK and control groups. A recent randomized prospective study showed that intensive blood glucose control with a target of 80 to 120 mg/dL during the early phase of ACS is associated with a marked reduction in platelet reactivity through multiple platelet signaling pathways.^27^ Our findings added additional support to the finding of this study and might suggest one physiological rationale to the use of GIK combined with intensive insulin therapy. Moreover, our study demonstrated that intensive insulin therapy resulted in an increase of fibrinolysis process, which is of great benefit for patients with hyperglycemia with ACS. Reduced fibrinolysis efficiency during hyperglycemia was previously explained by elevated PAI‐1.^28^ Chaudhuri et al^29^ demonstrated in the setting of ST‐segment elevation myocardial infarction that insulin infusion with maintenance of euglycemia exerted a profound profibrinolytic effect by suppression of PAI‐1 activity. What appears to be important in our findings is the fact that both surrogate outcome measures (PFA‐100 and PAI‐1) showed directional concordance and trends and add additional support to the utility of combining intensive glucose management to GIK therapy. This is especially interesting if we can ovoid severe hypoglycemia. The low hypoglycemia rate in our trial was remarkable and might be explained by the dedicated protocol with close glucose measurements. Of note, all the recorded hypoglycemic events in our study were not symptomatic and did not lead to the shutdown of the protocol.

## Study Limitations

We acknowledge several limitations of our study. First, it was conducted in a single center and lacks power to detect possible differences in individual primary outcomes, mainly deaths. Second, titrating appropriated doses of insulin is not an easy task, and patients are susceptible to hypoglycemia. Consequently, we had all of our nurses trained by a weeklong series of 1‐hour in‐service training sessions. All of them acquired good experience with the infusion protocols related to intensive insulin therapy. We acknowledge that it would not always be easy to apply this protocol in routine use by usual untrained nursing staff and untrained physicians. Third, this was not placebo‐controlled but rather an “open‐label” trial that could represent another potential bias. In this regard, the mechanistic underpinnings for the observed reduction in MACE in the GIKI_2_ group (PFA‐100 and PAI‐1 changes), together with the consistency of reduction in the 3 MACE components, provide substantial strength to our results. Fourth, the study may have benefitted from an additional group of intense insulin therapy on a placebo background to try to explain the mechanism underlying the beneficial effects of GIKI_2_. It was suggested that cardioprotective effects of a GIK cocktail are mainly mediated by insulin alone. We cannot rule out this possibility, and we are aware that our work did not get a clear answer to this important question.^30^ Last, the timing of the administration of GIK was not stated in the present study. This is an important factor as one of the central hypotheses was that major adverse clinical cardiovascular events are substantially reduced by GIK infusion when given early in the course of ACS.^9^ In our trial, timing of symptom onset was not available for a number of our patients since we did not predefine subgroups according to time from symptom onset to protocol treatment. It would be interesting to investigate this issue and assess the possible synergistic effect between early administration of GIK and intensive insulin therapy.

## Conclusions

This 1‐year analysis of the GIKI_2_ trial shows that in patients with non–ST‐segment elevation ACS, intensive insulin therapy combined with GIK seemed to reduce MACE compared with GIK alone or usual care. This beneficial effect might be related to the favorable action of glycemic control on platelet reactivity and fibrinolysis.

## Authors' Contributions

Dr Belguith performed statistical analysis; Drs Beltaief, Grissa, Msolli, and Boubaker handled funding and supervision; Drs Bzeouich, Sekma, and Echeikh, and M. Mzali acquired the data; Drs Nouira, Dridi, Khochtali, and Hassine conceived and designed the research; Dr Nouira drafted the article; and Drs Najjar, Bouida, and Boukef made critical revisions of the article for key intellectual content.

## Sources of Funding

This work was supported by the Tunisian Ministry of Higher Education and Scientific Research.

## Disclosures

None.

## Supporting information


**Appendix S1.** The Great Network Members.
