# Supplementary material for: One‐Year Outcome of Intensive Insulin Therapy Combined to Glucose‐Insulin‐Potassium in Acute Coronary Syndrome: A Randomized Controlled Study
Source: J Am Heart Assoc. 2017 Nov 14;6(11):e006674. doi: 10.1161/JAHA.117.006674 (PMC5721763; doi:10.1161/JAHA.117.006674)
Supplement: Supplementary file 1 — Appendix S1. The Great Network Members. [file JAH3-6-e006674-s001.pdf]

SUPPLEMENTAL MATERIAL

Appendix

The Great Network Members:

Alexandre Mebazaa, MD, PhD, Wahid Bouida, MD, Kaouthar Beltaief, MD, Mohamed Amine Msolli, MD, Nasri Bzeouich, MD, Adel Sekma, MD, Malek Echeikh, MD, Malek Mzali, MSC, Hamdi Boubaker, MD, Mohamed Habib Grissa, MD, Riadh Boukef, MD, Mohsen Hassine, MD, Zohra Dridi, MD, Asma Belguith, MD, Fadhel Najjar, MD, Ines Khochtali, MD, and Semir Nouira, MD.
